# Supplementary material for: Melanoma Detection by AFM Indentation of Histological Specimens
Source: Diagnostics (Basel). 2022 Jul 17;12(7):1736. doi: 10.3390/diagnostics12071736 (PMC9323377; doi:10.3390/diagnostics12071736)
Supplement: Supplementary file 1 [file diagnostics-12-01736-s001.zip › diagnostics-1767451-supplementary.pdf]

Supplementary Information

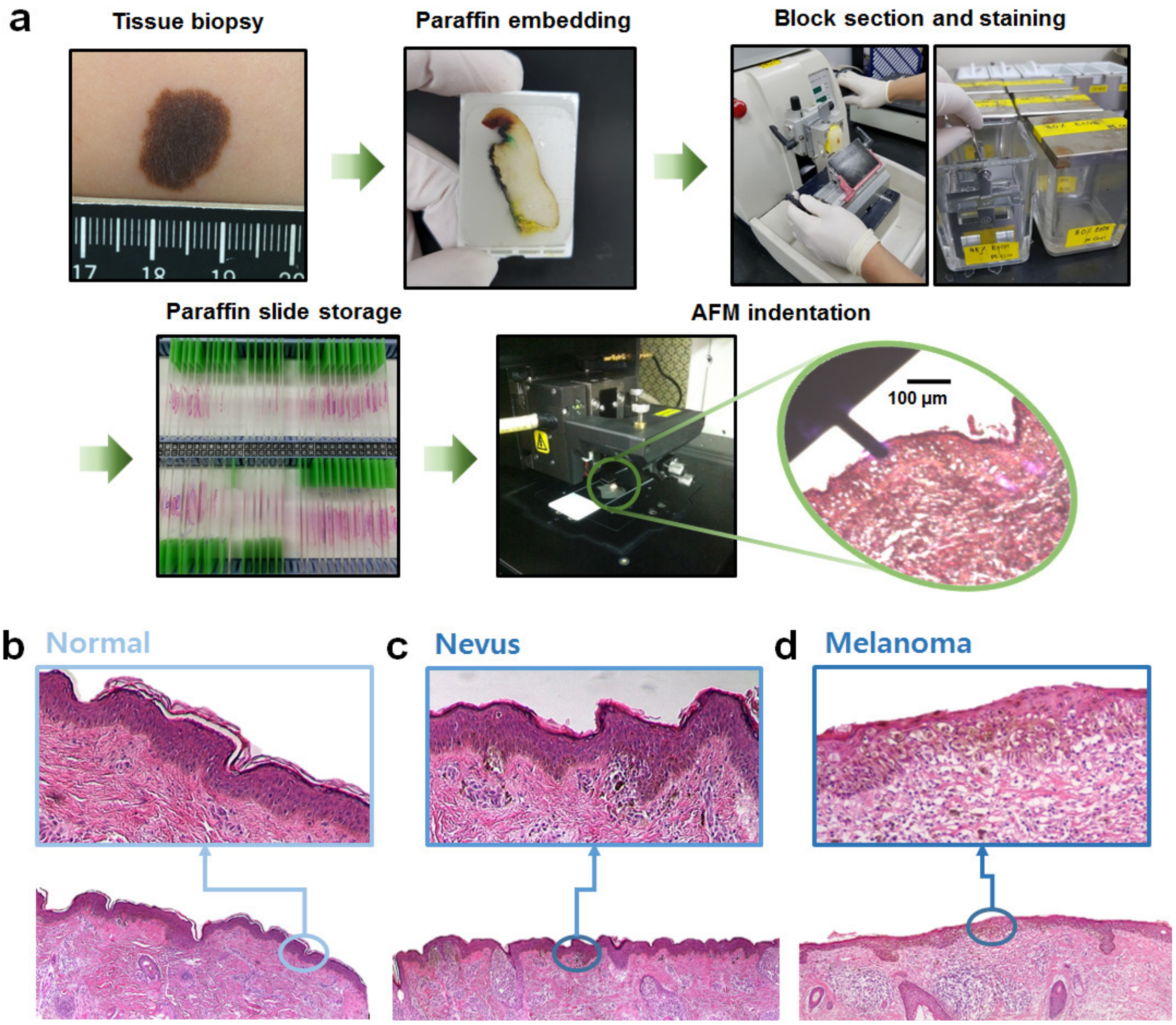

Figure. S1. (a) Preparation process of histological specimens and magnified optical images of H&E stained specimens of (b) normal, (c) benign nevus, and (d) melanoma.

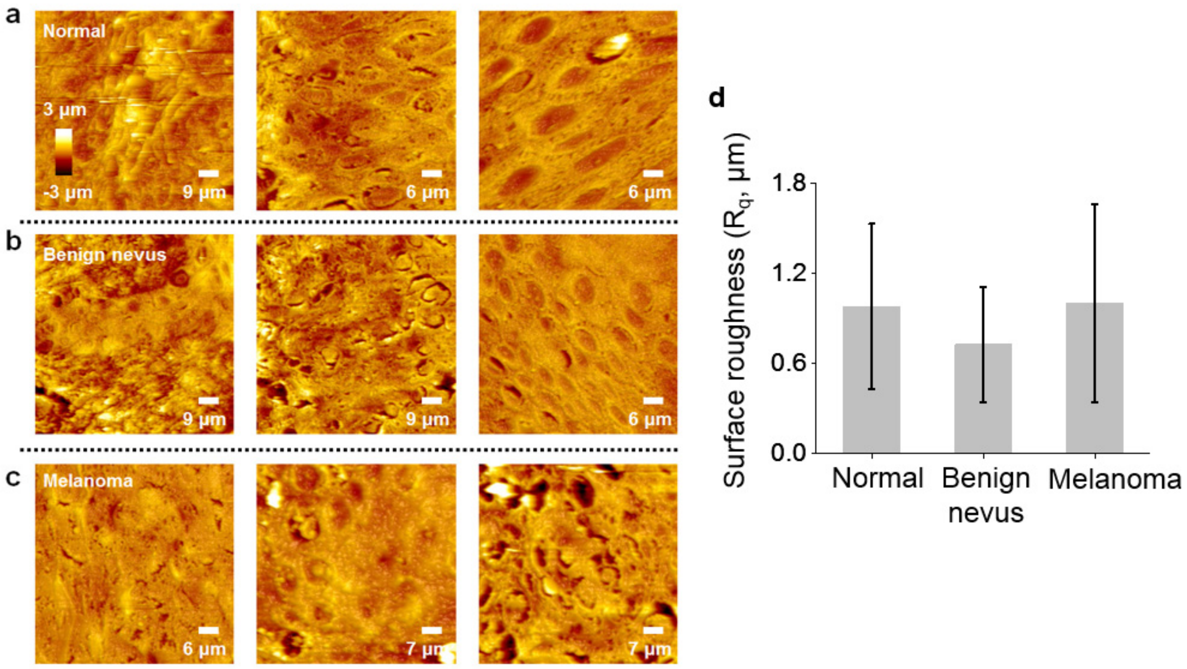

Figure. S2. AFM topographic images of (a) normal, (b) benign nevus, and (c) melanoma tissue specimen. AFM image size was 3,600–8,100 μm^2 depending on lesion sample. (d) The surface roughness (R_q) of normal, benign nevus, and melanoma sample, which was calculated by the following equation: $R_q = \sqrt{\frac{1}{n}\sum_{i=1}^{n} {y_i}^2}$.

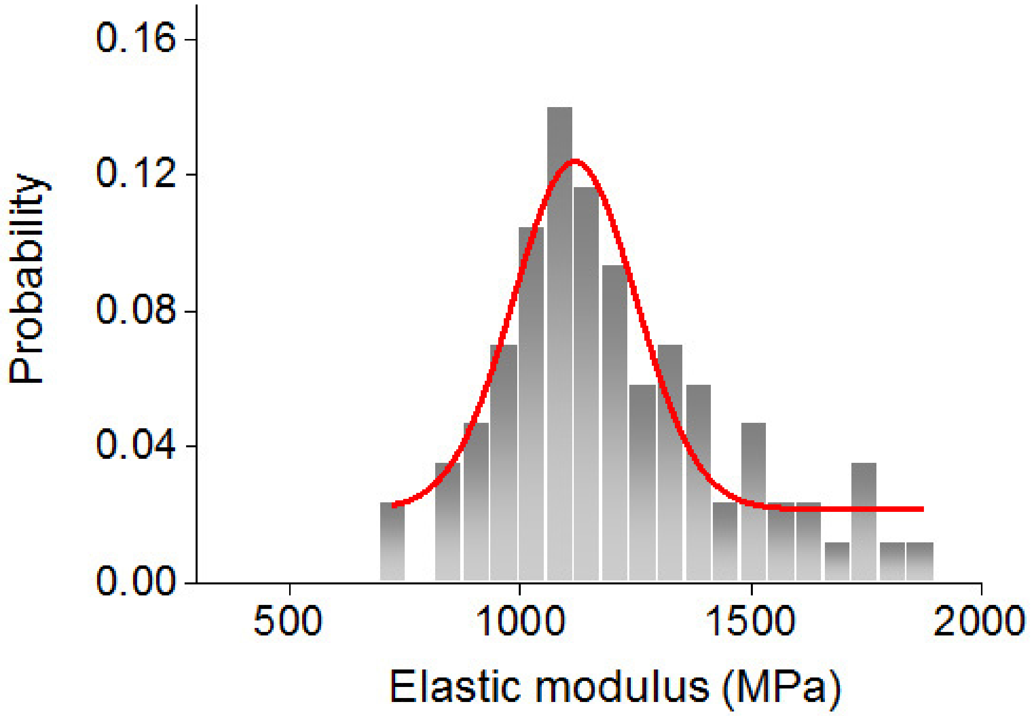

Figure. S3. Histogram of elastic modulus distribution of a glass substrate on which tissue specimens are placed. Elastic modulus was measured to be 1.1 ± 0.1 GPa. Note that the measured mechanical properties of glass substrate are not indicative of the intrinsic characteristic. Despite the fact, we came to know that the glass is always relatively more rigid than the sample.

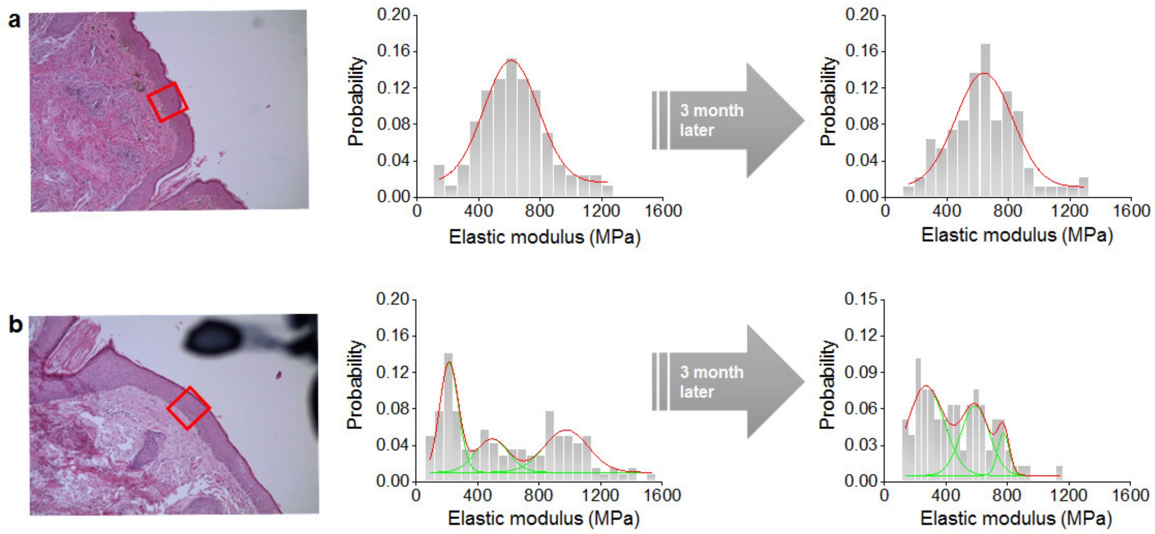

Figure. S4. Reproducibility of histological specimens for AFM indentation-based melanoma detection. The same lesion of each (a) benign nevus and (b) melanoma tissue specimen was probed after three months, showing that biomechanical signatures remained, even though it was hard to find the same exact spot that we probed three months ago.

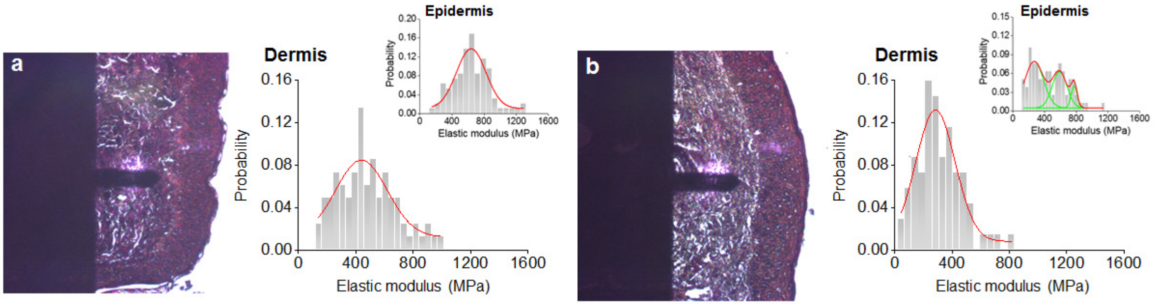

Figure. S5. Elastic modulus of histological specimen on dermis. Optical image and corresponding histogram of elastic modulus distribution of the lesion on (a) benign nevus and (b) melanoma in dermis. The inset histograms are results of the epidermis, showing difference from those of dermis.

Normal

No	Elastic modulus
1	415±221 MPa
2	401±148 MPa
3	398±252 MPa
4	439±101 MPa
5	521±153 MPa
6	437±261 MPa
7	370±105 MPa

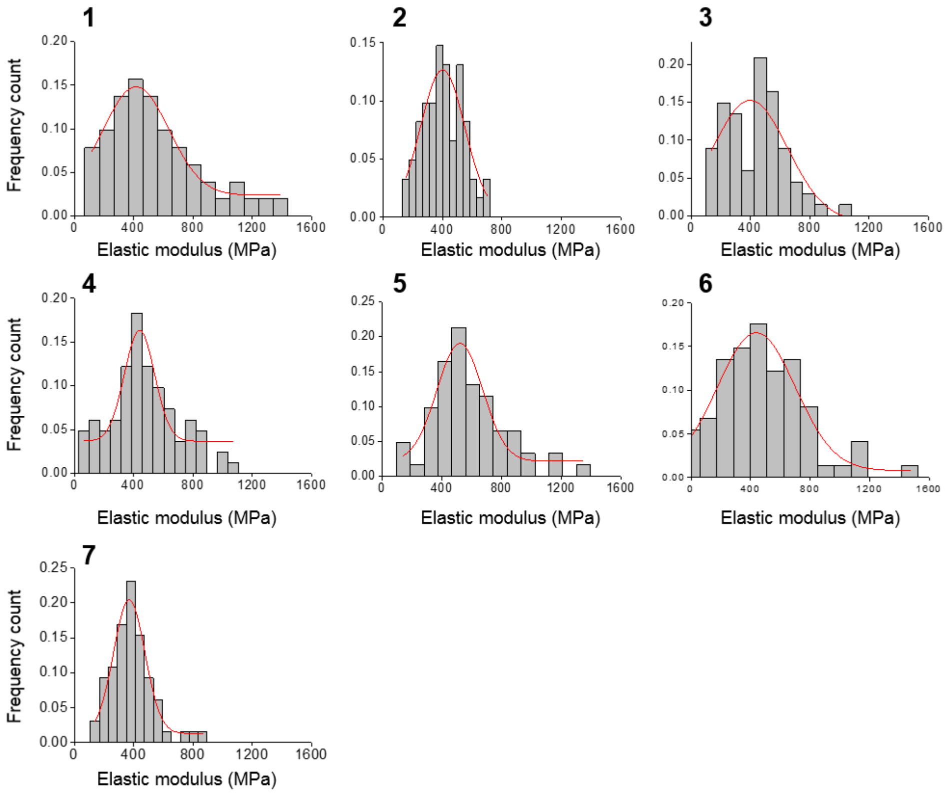

Figure. S6. Biomechanical signatures of healthy epidermal skin tissues characterized by unimodal distribution with average single-peak stiffness from 398 to 521 MPa.

Nevus

No	Elastic modulus
1	485±179 MPa
2	450±182 MPa
3	467±178 MPa
4	441±164 MPa
5	575±107 MPa
6	505±162 MPa
7	611±217 MPa
8	848±299 MPa
9	618±187 MPa
10	636±203 MPa
11	609±176 MPa
12	650±156 MPa
13	783±177 MPa

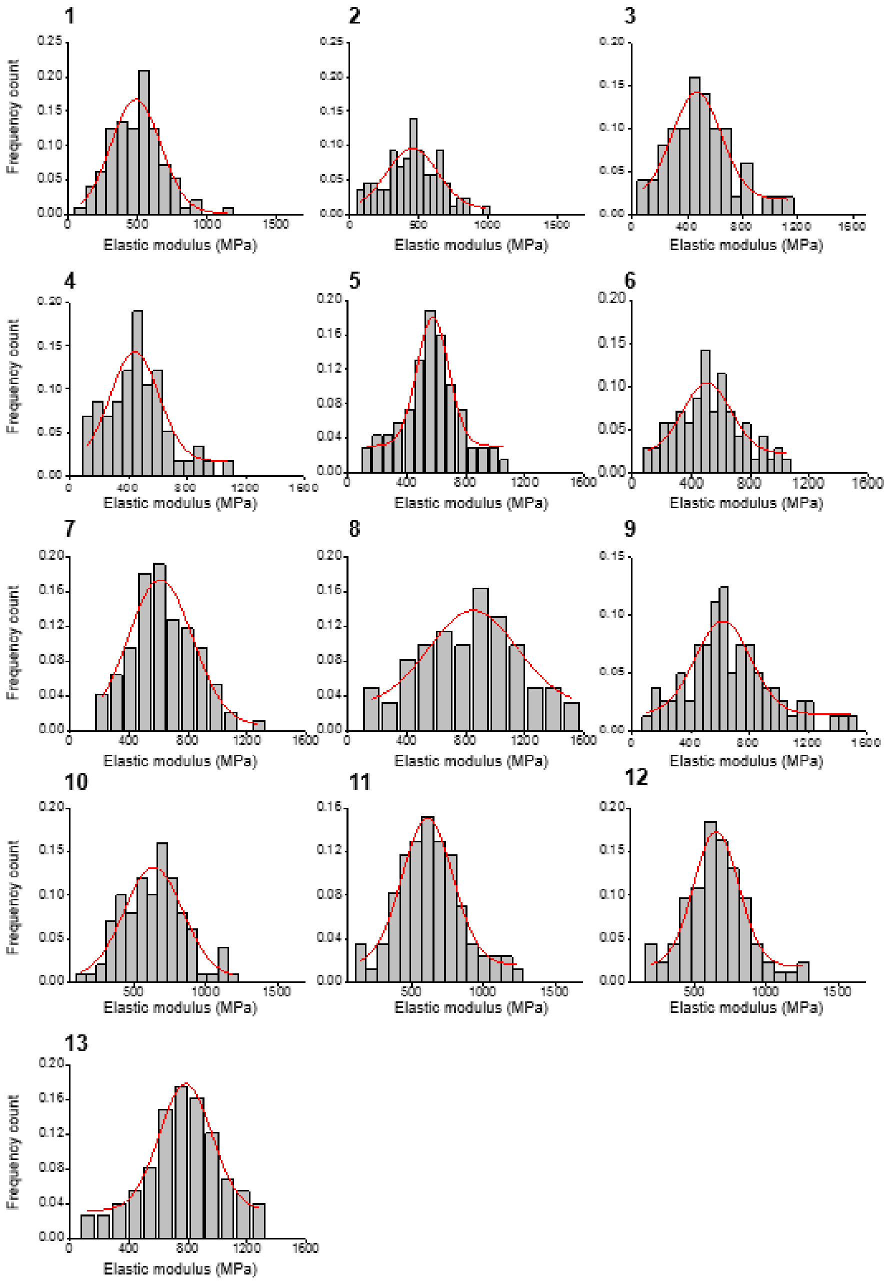

Figure. S7. Benign nevi tissues with different biomechanical signatures. All histograms of elastic modulus distributions exhibited single-peak distribution. Elastic modulus of cases 1 to 6 were similar to those of normal tissues, whereas elsctic mosulus of the cases 7 to 13 were generally 100 MPa higher than those of cases 1 to 6 (see Table 1 in the text).

Melanoma

No	Elastic modulus		
1	230±49 MPa	483±97 MPa	782±59 MPa
2	158±92 MPa	484±88 MPa	861±58 MPa
3	272±95 MPa	542±80 MPa	893±63 MPa
4	188±79 MPa	497±110 MPa	787±56 MPa
5			667±221 MPa
6	211±62 MPa	400±47 MPa	606±19 MPa
7		373±139 MPa	725±55 MPa
8	274±93 MPa	475±57 MPa	649±39 MPa
9	219±73 MPa	396±43 MPa	610±146 MPa
10	212±40 MPa	486±91 MPa	
11		363±117 MPa	611±39 MPa
12	227±55 MPa		612±111 MPa
13	193±27 MPa	387±49 MPa	767±28 MPa

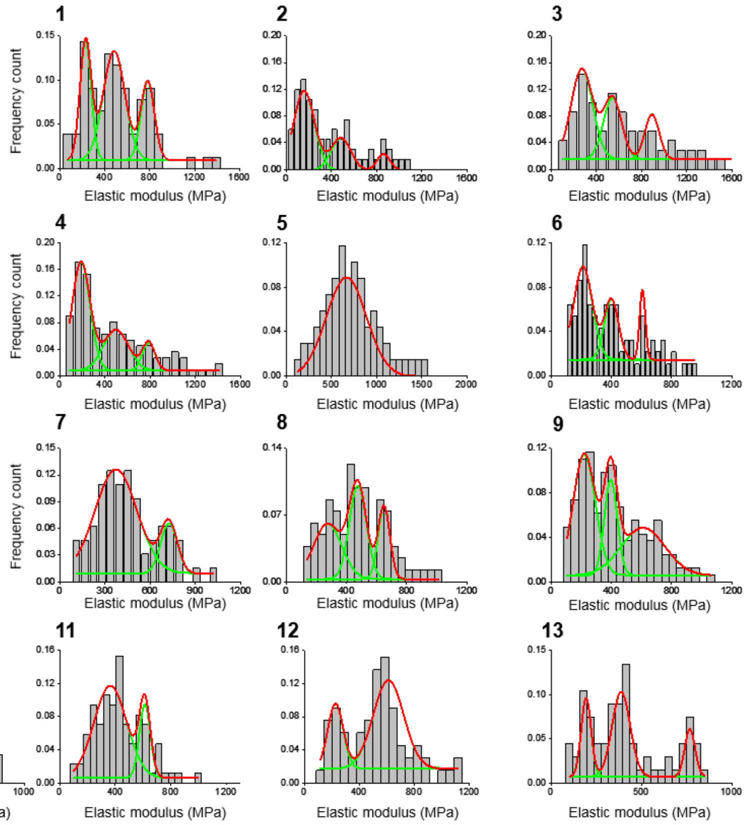

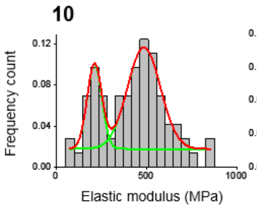

Figure. S8. Stiffness profiles of melanoma tissues revealing distinct phenotype in biomechanical properties. Histograms exhibited three multi-peak distributions ranging from the 1st to the 3rd peak region in the melanoma tissues, particularly in early melanoma tissues (Clark level I). However, this phenomenon was found to be occasionally unsustainable depending on cancer stage. The soft tissue properties in cases 5, 7, and 11 disappeared whereas such rigid properties rarely disappeared in cases 10. Case 5 showed single-peak distribution characteristics in elasticity. The complexity is attributed to heterogeneities of melanoma tissues.

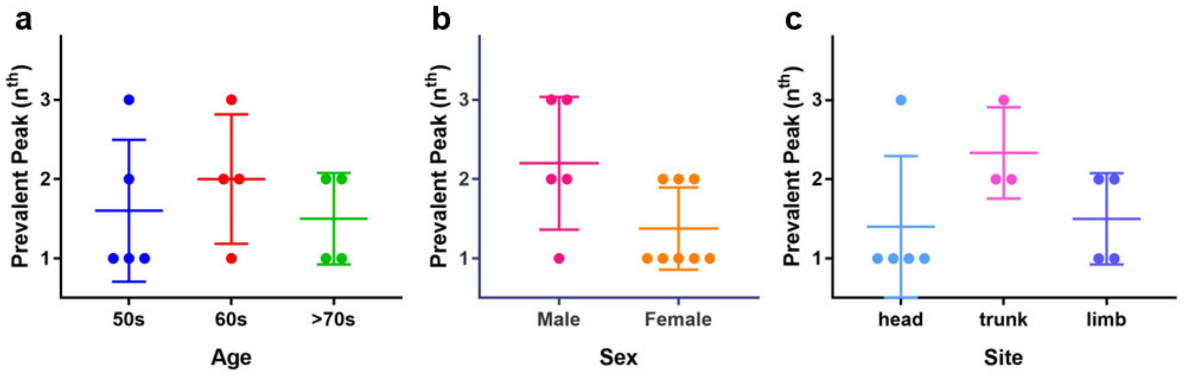

Figure. S9. The most prevalent peak (P_m) — one with the highest population — among multiple peaks on melanoma tissues depending on age (a), sex (b), and site (c). For all items in each graph, Student's t-test results did not show significant difference (P-value> 0.05).
